# Multiple Cold Abscesses of a Chest Wall with Ribs Destruction in a Hemodialysis Patient

**DOI:** 10.3390/reports7010009

**Published:** 2024-02-01

**Authors:** Chih-Chun Kuo, Po-Jen Hsiao, Tai-You Kuo, Wen-Fang Chiang

**Affiliations:** 1 Division of Endocrinology and Metabolism, Department of Medicine, Armed Forces Taoyuan General Hospital, Taoyuan 325011, Taiwan; lg1001010@gmail.com; 2 Division of Nephrology, Department of Medicine, Armed Forces Taoyuan General Hospital, Taoyuan 325011, Taiwan; a2005a660820@yahoo.com.tw; 3 Division of Nephrology, Department of Medicine, Tri-Service General Hospital, National Defense Medical Center, Taipei 114202, Taiwan; 4 Division of Hematology and Oncology, Department of Medicine, Armed Forces Taoyuan General Hospital, Taoyuan 325011, Taiwan; yow623@yahoo.com.tw

**Keywords:** abscess, catheter, chest wall, hemodialysis, *Staphylococcus aureus*, thrombophlebitis

## Abstract

Cold abscess of the chest wall is an uncommon disease that is mainly caused by tuberculous infection. Staphylococcal cold abscesses of the chest wall are extremely rare and usually clinically occult, frequently leading to a delay in diagnosis and consequently severe infectious complications. We report an 88-year-old woman with end-stage renal disease, who presented with an exit site infection of a cuffed tunneled dialysis catheter caused by methicillin-resistant *Staphylococcus aureus* (MRSA). Despite the removal of the catheter and administration of antibiotics, she developed refractory MRSA bacteremia. Computed tomography (CT) of the chest revealed septic thrombophlebitis with metastatic cold abscesses of the chest wall and ribs destruction. Although CT-guided drainage and vancomycin therapy eliminated MRSA bacteremia, the chest wall abscesses did not resolve. Patients fitted with a central venous dialysis catheter are at risk of septic thrombophlebitis with metastatic cold abscesses of the chest wall that are resistant to antibiotic therapy. Early identification is based on serial blood cultures and prompt CT scans. Surgical management coupled with antibiotic therapy can eradicate the source of infection and improve patients’ outcomes.

## 1. Introduction

In hemodialysis patients whose arteriovenous access is immature or cannot be readily created, a cuffed tunneled dialysis catheter is used for vascular access. Although a cuff bonded to the catheter can prevent bacterial migration from the patient’s skin, the risk of exit site and tunnel infection and bacteremia increases with the duration of catheterization. Because the patient will continue to require dialysis treatment, removal of the catheter can be delayed in the absence of hemodynamic instability and septic signs [1]. However, during the salvage of an infected catheter, metastatic infections, including septic thrombophlebitis, endocarditis, osteomyelitis and spinal epidural abscess may occur, albeit rarely [2,3].

Chest wall abscesses can occur primarily as a result of hematogenous spread from a distant infected tissue, as a consequence of trauma or surgery to the region, or through contiguous pulmonary or pleural infection [4]. In contrast to the typical signs of inflammation in hot abscesses, a cold abscess is not accompanied by redness, tenderness or fever [5]. Although cold abscesses of the chest wall can be observed in immunocompromised patients, they are an extremely rare complication from a cuffed tunneled dialysis catheter. We describe an 88-year-old woman who presented with an infection of the exit site of a cuffed tunneled hemodialysis catheter. Persistent bacteremia following the removal of the catheter led to the diagnosis of septic thrombophlebitis complicated by the presence of metastatic cold abscesses of the chest wall and associated ribs destruction.

## 2. Detailed Case Description

An 88-year-old woman presented to the emergency department with a fever and dyspnea. Eight months before the current admission, the patient was diagnosed with end-stage renal disease, requiring regular hemodialysis through a cuffed tunneled double-lumen catheter in the right internal jugular vein. She began experiencing cough, fatigue, weakness and poor appetite one week before the current admission. She had presented to the same hospital and was found to have a white-cell count of 11,330/mm^3^ (normal range, 4500–11,000). She was diagnosed with acute bronchitis and discharged with prescription medication. One day before the current admission, she was seen by a local doctor with complaints of increasing fatigue, fever and dyspnea, and she was transferred to the hospital. There was no sore throat, chest wall pain, nausea, vomiting, diarrhea or abdominal pain. Her past medical history included hypertension, type 2 diabetes mellitus with diabetic kidney disease and ischemic stroke with right hemiparesis. Her medications included aspirin, sitagliptin, insulin, irbesartan, nifedipine extended release, folic acid, vitamin B complex, calcium carbonate and epoetin beta. The patient’s family history was unremarkable.

On examination, her temperature was 38.7 °C, blood pressure 101/51 mmHg, pulse 88/min, respiratory rate 22/min and oxygen saturation 100%, while she was receiving oxygen through a simple face mask at a rate of 6 L/min. Her level of consciousness was confused. Results of the physical examination were notable for erythema and a purulent discharge at the exit site of the dialysis catheter. The appearance of her tonsils and oropharynx were normal and there was no heart murmur or mass of the chest wall. Laboratory studies showed a white cell count of 17,740/mm^3^ (normal range, 4500–11,000), C-reactive protein 36.1 mg/dL (normal range, <0.5), and albumin 2.71 g/dL (normal range, 3.1–4.8) (Table 1). Dipstick urinalysis was positive for protein and negative for blood, and a microscopic examination showed no sediment. A chest X-ray revealed an enlargement of the cardiac silhouette. An electrocardiogram revealed a sinus rhythm and no evidence of myocardial ischemia or infarction.

**Figure 1:**
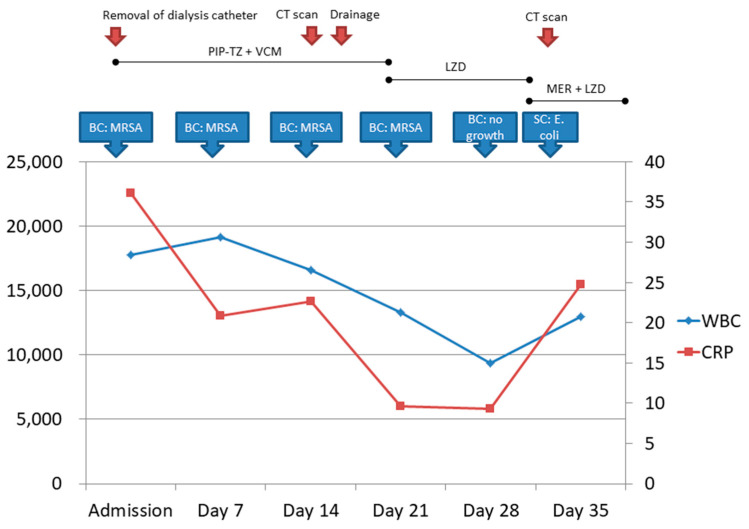
Hospital course. Abbreviations: BC, blood culture; CT, computed tomography; CRP, C-reactive protein; LZD, linezolid; MER, meropenem; MRSA, methicillin-resistant *Staphylococcus aureus*; PIP-TZ, piperacillin/tazobactam; SC, sputum culture; VCM, vancomycin; WBC, white cell count.

**Figure 2:**
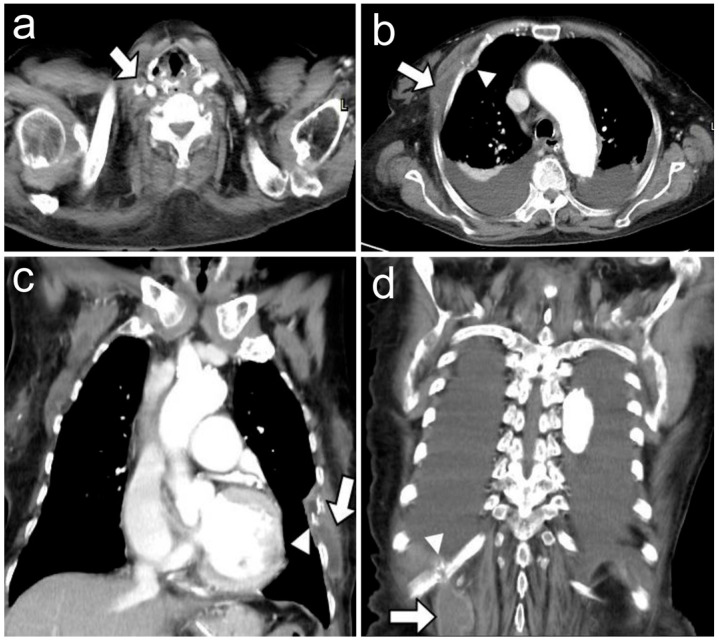
Contrast-enhanced computed tomography of the chest shows a filling defect in the right internal jugular vein (arrow) (**a**) and three low-density lesions with rim enhancement in right pectoralis major muscle (arrow) with destruction of right anterior third rib (arrowhead) (**b**), left chest wall (arrow) with destruction of left sixth rib (arrowhead) (**c**), and right posterior chest wall (arrow) with destruction of right eleventh rib (arrowhead) (**d**).

Following the removal of the dialysis catheter, a regimen of intravenous vancomycin plus piperacillin/tazobactam was initiated (Figure 1). Both blood and catheter tip cultures yielded methicillin-resistant *Staphylococcus aureus* (MRSA, vancomycin minimum inhibitory concentration (MIC) 1 μg/mL and linezolid MIC 2 μg/mL). Transthoracic echocardiography disclosed no evidence of valvular vegetation. Despite vancomycin treatment for more than two weeks, the patient continued to have a fever accompanied by MRSA bacteremia. Computed tomography (CT) of the chest and abdomen revealed a filling defect in the right internal jugular vein and multiple rim enhancement fluid collections in the chest wall together with bone destruction of the right third and eleventh and left sixth ribs (Figure 2). Taken together, this information suggested septic thrombosis of the right internal jugular vein and abscesses of the chest wall associated with rib destruction. Because the patient was considered to be at high risk, her family members decided against surgical intervention. CT-guided drainage of the subcutaneous abscesses was performed under local anesthesia. About 50 mL of purulent fluid was drained and the culture subsequently also grew MRSA. Acid-fast stain and fungus culture of the pus specimen revealed negative results. Subsequent blood cultures also revealed the growth of MRSA (vancomycin MIC 1 μg/mL and linezolid MIC 2 μg/mL). The antibiotic regimen was switched to linezolid (Figure 1).

After four weeks of hospitalization, her white cell count decreased to 9370/mm^3^ and the C-reactive protein level decreased to 9.3 mg/dL. Furthermore, blood culture showed no bacterial growth on the 28th day of hospitalization (Figure 1). She did not report chest wall pain during the course of hospitalization. Despite these improvements, she experienced a recurrence of fever accompanied by dyspnea. A follow-up CT scan of the chest revealed the persistence of the three chest wall abscesses with no change in their size. Her family member refused invasive treatment. We changed antibiotic coverage to meropenem and linezolid. Unfortunately, the patient died of hospital-acquired pneumonia caused by bilateral infection of the lower lungs with multidrug-resistant *Escherichia coli* five weeks after admission.

## 3. Discussion

We present the case of a patient with immune suppression (due to age, diabetes, chronic kidney disease and hemodialysis) in whom a common infection occurred (infection of the jugular vein catheter) with a rare spread by the bloodstream to the chest wall and ribs that may potentially damage respiratory mechanics if left untreated. Central venous catheters are the most common cause of internal jugular vein thrombosis [6]. Vessel wall damage, flow disruption and the tip of the catheter may augment clot formation. MRSA colonization over the catheter tip could result in septic thrombophlebitis. In the case presented here, the patient’s thrombophlebitis was diagnosed on the basis of radiographic evidence on contrast-enhanced CT and because of persistent MRSA bacteremia despite vancomycin therapy. The infected thrombus served as a nidus from which bacteria spread via the bloodstream to the distant site. The patient did not have a history of trauma or surgery to the chest wall, and there was no underlying pulmonary or pleural infection on presentation. The abscesses of the chest wall are a complication of hematogenous spread of MRSA from an infected dialysis catheter and thrombophlebitis.

One may argue that the patient could have been diagnosed with cuffed catheter-related Lemierre’s syndrome, which has been reported in a hemodialysis patient [7]. Lemierre’s syndrome is characterized by thrombophlebitis of the internal jugular vein and septicemia caused by infection in the head and neck area accompanied by distal metastatic infections, especially of the lungs and joints [8]. The primary source is mostly oropharyngeal infection, such as pharyngitis or tonsillitis, although otitis media, mastoiditis, sinusitis and dental infection have also been reported. The most common pathogen is anaerobic *Fusobacterium necrophorum* and others include *Fusobacteria* spp, *Klebsiella pneumoniae*, *Pseudomonas aeruginosa*, *Staphylococcus aureus* and *Streptococci* [8]. While the criteria for diagnosis of Lemierre’s syndrome have varied over time [9], strictly speaking, our patient could not have been diagnosed with Lemierre’s syndrome because there was no evidence of oropharyngeal infection and central venous dialysis catheters often provide a route whereby organisms can penetrate intact skin and induce infection.

Cold abscesses are most often caused by tuberculous infection, which is a rare form of extrapulmonary tuberculosis. Non-tuberculosis cold abscesses are rare and can be caused by both fungal and bacterial infections. In fungal infection, coccidioidomycosis, actinomycosis, blastomycosis, histoplasmosis sporotrichosis and chromomycosis have been reported [10]. Bacterial infections that induce cold abscesses include *Staphylococcus aureus* infections, melioidosis and mixed bacterial infections [5,11,12]. Job’s syndrome, also called hyper-IgE syndrome, is a rare primary immunodeficiency characterized by severe eczematous skin eruptions, recurrent staphylococcal cold abscesses, recurrent pulmonary infections and high levels of serum IgE [13]. In the present case, the patient did not have the clinical manifestations of Job’s syndrome, making the diagnosis of this disease unlikely. The patient’s compromised immunity leading to cold abscesses of the chest wall was most likely due to old age together with her underlying uremia and type 2 diabetes.

*Staphylococcus aureus* infection is well-known for its pyogenic pathogenesis, causing both local and metastatic abscesses, particularly in vulnerable hemodialysis patients. By contamination, colonization or biofilm formation on tissue surfaces, these organisms could invade the host, establish localized infection, escape from the immune response and metastasize. Among *Staphylococcus aureus* infections, catheter-related bloodstream infections (CRBSIs) pose a significant concern for hemodialysis patients relying on vascular catheters for access. These infections occur when bacteria enter the bloodstream through the catheter site. Hemodialysis patients are particularly vulnerable due to the repeated use of catheters for vascular access, and CRBSIs can lead to serious complications such as sepsis, a severe, life-threatening condition that can result in organ dysfunction and failure. CRBSIs may also lead to infectious complications in distant organs, including infective endocarditis, osteomyelitis, septic arthritis, spinal epidural abscess and septic emboli, further complicating the management of these infections [2,14,15]. Additionally, CRBSIs can contribute to the development of septic thrombophlebitis, which may lead to catheter dysfunction, diminishing the effectiveness of hemodialysis. The repeated occurrence of CRBSIs can compromise the longevity of vascular access, limiting future dialysis options for the patient. Metastatic infections in our patient include septic thrombophlebitis and chest wall abscesses. Although infective endocarditis is a possibility, our patient did not undergo transesophageal echocardiography for diagnosis due to the family’s decision against the invasive procedure. Persistent MRSA bacteremia has necessitated a prolonged course of antibiotic therapy, contributing to the development of antibiotic-resistant *Escherichia coli* pneumonia, ultimately leading to the patient’s demise. In addition to chest wall abscesses, our patient also showed bone destruction of the ribs. It has been reported that staphylococcal cold abscess of the chest wall with rib destruction can be secondary to tricuspid endocarditis [16]. Intracellular infection can either induce bone destruction or provide reservoirs for infection recurrence [17]. Because MRSA bacteremia was eliminated, the persistent chest wall abscesses may have resulted from the spread of *Staphylococcus aureus* from the ribs.

Staphylococcal cold abscesses of the chest wall are rare and early detection and timely intervention are often challenging. Das et al. described a case of a 50-year-old immunocompetent man with a staphylococcal cold abscess in the anterior chest wall. The diagnosis was based on asymptomatic and progressively enlarging soft tissue swellings over one month [12]. Pichon et al. also documented a case of staphylococcal cold abscess with rib destruction in the anterior chest wall, involving a 25-year-old intravenous drug user who was diagnosed due to soft tissue swellings with insidious onset and progressive enlargement over one week [16]. As shown in our patient, the relatively small size of the abscesses and the absence of inflammation led to a delay in diagnosis. Even so, their damaging effects on respiratory mechanics and their potential for dissemination into the pleural cavity and mediastinum can lead to devastating consequences that are potentially life threatening [18]. In addition to detailed physical examinations and serial blood cultures, a contrast-enhanced CT scan can help in the early diagnosis of cold abscesses of the chest wall, especially in an immunocompromised patient [19]. Persistent MRSA bacteremia, despite the administration of appropriate antibiotics, together with a timely CT scan, identified the cold abscesses of the chest wall in our patient.

Treatment of septic thrombophlebitis includes removal of the central venous catheter and broad-spectrum antimicrobial administration, as well as possible surgical excision [20]. Although Staphylococcal cold abscesses of the chest wall are rarely reported and there is limited therapeutic experience, the development of a treatment plan can be based on similar diseases. As shown in our patient, antibiotic administration together with drainage of the abscesses failed to improve the outcome. Others have suggested that tuberculous cold abscesses of the chest wall should be treated by complete resection coupled with anti-tuberculous administration [21]. The two aforementioned cases reported by Das and Pichon et al. were successfully treated with antistaphylococcal antibiotics, coupled with incision and drainage, resulting in complete recovery. The favorable outcome observed in these patients could be attributed to their younger age and the implementation of surgical incision and drainage. In bacterial abscesses of the chest wall, urgent and complete surgical debridement of the infected tissue combined with antibiotic therapy is the mainstay of treatment [22]. Because adverse effects on respiratory mechanics may result from aggressive surgical intervention, negative-pressure wound therapy or chest wall reconstruction is a good option for wound management [4]. Importantly, persistent MRSA bacteremia is linked to heightened morbidity and mortality. In addition to source control, shifting from vancomycin to an alternative regimen or adopting combination therapy, such as daptomycin, linezolid, or ceftaroline, may enhance patient outcomes [23].

## 4. Conclusions

Septic thrombophlebitis of the internal jugular vein with metastatic cold abscesses of the chest wall and ribs destruction is a rare complication of cuffed tunneled dialysis catheterization. A lack of inflammatory signs makes early diagnosis of cold abscesses of the chest wall difficult and this can potentially be fatal. Serial blood cultures and timely radiographic imaging are key to prompt recognition and treatment of this condition.

## Figures and Tables

**Table 1:** Laboratory data on admission.

Tests	Normal Range	Patient’s Values
White cell count	4500–11,000/mm^3^	17,740
Hemoglobin	12–16 g/dL	10.9
Platelets	130,000–400,000/mm^3^	204,000
Blood urea nitrogen	6–24 mg/dL	68.3
Creatinine	0.5–1.4 mg/dL	7.11
Aspartate aminotransferase	10–30 U/L	29
Alanine aminotransferase	2–32 U/L	28
Sodium	137–145 mmol/L	141
Potassium	3.1–5.3 mmol/L	4.1
Calcium	8.8–10.6 mg/dL	7.4
Phosphate	2.6–4.4 mg/dL	4.2
C-reactive protein	<0.5 mg/dL	36.1
Albumin	3.1–4.8 g/dL	2.71
pH	7.35–7.45	7.433
PaO_2_	75–100 mmHg	104.1
PaCO_2_	35–45 mmHg	31.5
HCO_3_^−^	22–26 mmol/L	21.3
O_2_ saturation	92–98.5%	99.8

## Data Availability

Data are contained within the article.
